# Genetically Predicted Telomere Length and Its Relationship With Alzheimer’s Disease

**DOI:** 10.3389/fgene.2021.595864

**Published:** 2021-02-19

**Authors:** Guangping Yu, Leihong Lu, Zaihong Ma, Shouhai Wu

**Affiliations:** ^1^Wuqing Center for Disease Control and Prevention, Tianjin, China; ^2^Linyi People’s Hospital, Linyi, China; ^3^Sun Yat-sen Memorial Hospital, Sun Yat-sen University, Guangzhou, China; ^4^The Second Affiliated Hospital of Guangzhou University of Chinese Medicine, Guangzhou, China

**Keywords:** telomeres, Alzheimer’s disease, Mendelian randomization, epidemiology, risk factors

## Abstract

Are shorter telomeres causal risk factors for Alzheimer’s disease (AD)? This study aimed to examine if shorter telomeres were causally associated with a higher risk of AD using Mendelian randomization (MR) analysis. Two-sample MR methods were applied to the summary effect sizes and standard errors from a genome-wide association study for AD. Twenty single nucleotide polymorphisms of genome-wide significance were selected as instrumental variables for leukocyte telomere length. The main analyses were performed primarily using the random-effects inverse-variance weighted method and complemented with the other three methods: weighted median approaches, MR-Egger regression, and weighted mode approach. The intercept of MR-Egger regression was used to assess horizontal pleiotropy. We found that longer telomeres were associated with lower risks of AD (odds ratio = 0.79, 95% confidence interval: 0.67, 0.93, *P* = 0.004). Comparable results were obtained using weighted median approaches, MR-Egger regression, and weighted mode approaches. The intercept of the MR-Egger regression was close to zero. This may show that there was not suggestive of horizontal pleiotropy. Our findings provided additional evidence regarding the putative causal association between shorter telomere length and the higher risk of AD.

## Introduction

Telomeres are composed of nucleotides and proteins, which are found at the end of each chromosome. Their functions are assumed to be stabilizing the structures of chromosomes and protecting the end of chromosomes from fusion with adjacent ones (Blackburn et al., 2015). Although dementia has unknown causes (Boyle et al., 2019; Morbelli et al., 2019; Ohara et al., 2019; Tropea et al., 2019; George et al., 2020; Iversen et al., 2020; Lu et al., 2020; Martinez-Miller et al., 2020; Mueller et al., 2020; Nadim et al., 2020; Osler et al., 2020; Scarioni et al., 2020; Seblova et al., 2020; Strandberg et al., 2020), several previous studies, including epidemiological surveys and clinical reports, showed that telomeres were shorter in patients with Alzheimer’s disease (AD), cognitive disorders, other aging-related diseases, and mortality (Panossian et al., 2003; Franco et al., 2006; Thomas et al., 2008; Lukens et al., 2009; Zekry et al., 2010; Guan et al., 2012; Hochstrasser et al., 2012; Honig et al., 2012; Moverare-Skrtic et al., 2012; Takata et al., 2012; Mathur et al., 2014; Kota et al., 2015; Tedone et al., 2015; Zhan et al., 2015, 2018a,b; Staffaroni et al., 2018; Wang et al., 2018; Zhan and Hagg, 2018, 2020; Gao et al., 2019; Guo and Yu, 2019; Kaja et al., 2019; Subedi et al., 2019; El Assar et al., 2020; Fani et al., 2020). A recent meta-analysis that summarized these findings (Panossian et al., 2003; Franco et al., 2006; Thomas et al., 2008; Lukens et al., 2009; Zekry et al., 2010; Guan et al., 2012; Hochstrasser et al., 2012; Honig et al., 2012; Moverare-Skrtic et al., 2012; Takata et al., 2012; Mathur et al., 2014; Kota et al., 2015; Tedone et al., 2015; Fani et al., 2020; Supplementary Table 1) concluded that shorter telomeres were associated with higher risks of AD (Forero et al., 2016; Smith et al., 2019). Of them, only two used prospective cohort designs and studied incident AD cases. The others, however, used case–control designs. These studies, taken together, provided valuable information regarding the roles of telomeres in AD. However, significant limitations as acknowledged in these publications are also obvious—small sample sizes in these case–control studies or prospective cohort investigations and residual confounding that was not collected in these studies. All these limitations render it hard to make firm conclusions on if telomere length is a potential causal risk factor or merely a predictive biomarker for AD.

To address concerns of unmeasured confounding (e.g., unmeasured shared environmental factors) and to exploit the large sample sizes for this type of aim, Mendelian randomization (MR) design using two samples was previously discussed. MR methods make the best use of genetic variants as instrumental variables to test if there is an association between an exposure and an outcome and further to calculate the effect magnitude of an exposure variable on an outcome variable (Smith and Ebrahim, 2004). This approach has been used to examine these topics previously (Zhan et al., 2015; Zhan and Hagg, 2018; Gao et al., 2019; Guo and Yu, 2019), which found that shorter telomeres were associated with higher AD risks. However, a recent study did not find a significant association between telomeres and Parkinson’s disease (Chen and Zhan, 2020). In the present short communication, we aimed to revisit the relationship between telomere length and AD using the MR design. We will use the updated summary statistics generated previously from the published genome-wide association study (GWAS) for leukocyte telomere length (Li et al., 2020) and clinically diagnosed AD (Kunkle et al., 2019).

## Materials and Methods

### Instrument Variable Selection

In the most recent GWAS of leukocyte telomere length (Li et al., 2020), the European Network for Genetic and Genomic Epidemiology^1^ conducted a GWAS for leukocyte telomere length in 78,592 individuals of European ancestry. Mean leukocyte telomere length measurements were conducted using an established quantitative polymerase chain reaction technique, which expressed telomere length as a ratio of the telomere repeat number (T) to a single-copy gene (S). Leukocyte telomere length measurements were standardized either by using a calibrator sample or quantifying against a standard curve. In total, 20 single-nucleotide polymorphisms (SNPs) at 17 genomic loci were selected. These SNPs were reported to be independently top loci for leukocyte telomere length, and they were of genome-wide statistical significance (at a level: *P* < 5 × 10^–8^). In our study, we use these 20 SNPs as instrumental variables and included proxy SNPs through LDlink if SNPs were unavailable in the AD GWAS or found to be palindromic. These variants explained around a 2% variance of leukocyte telomere length. These SNPs are presented in Table 1.

**TABLE 1:** SNPs selected as instrumental variables and their associations with leukocyte TL in European Network for Genetic and Genomic Epidemiology Consortium and with AD in International Genomics of Alzheimer’s Project Consortium.

SNP	Chromosome	Position (hg37)	Effect allele	Other allele	β_TL	se(β_TL)	β_AD	se(β_AD)
rs3219104	1	226562621	C	A	0.041	0.006	0.021	0.019
rs55749605	3	101232093	A	C	–0.037	0.006	0.019	0.014
rs2293607	3	169482335	C	T	–0.086	0.006	0.019	0.016
rs13137667	4	71774347	C	T	0.076	0.013	–0.055	0.038
rs2086240	4	164098317	G	T	0.055	0.005	–0.027	0.017
rs7705526	5	1285974	A	C	0.081	0.005	–0.034	0.018
rs2853677	5	1287194	A	G	–0.063	0.005	0.011	0.016
rs34991172	6	25480328	G	T	–0.060	0.010	–0.013	0.031
rs707919	6	31641139	G	A	0.033	0.005	–0.014	0.015
rs59294613	7	124554267	A	C	–0.040	0.005	0.008	0.015
rs9419958	10	105675946	C	T	–0.063	0.007	0.015	0.020
rs228595	11	108105593	A	G	–0.028	0.005	–0.008	0.014
rs2286836	14	73442192	T	C	0.045	0.008	–0.035	0.023
rs7194734	16	82199980	T	C	–0.036	0.006	−2.00E-04	0.016
rs3785074	16	69406986	G	A	0.035	0.005	–0.002	0.016
rs62053580	16	74680074	G	A	–0.038	0.007	0.030	0.021
rs8105767	19	22215441	G	A	0.039	0.005	–0.011	0.015
rs75691080	20	62269750	T	C	–0.067	0.009	–0.040	0.026
rs73624724	20	62436398	C	T	0.050	0.007	−8.00E-04	0.021
rs13038527	20	62218340	A	G	–0.138	0.022	0.070	0.063

### Assumptions of Mendelian Randomization Design

Three assumptions are needed for the MR analysis. Firstly, the instrumental variables are robustly associated with the exposure of interest (i.e., telomere length). Secondly, instrumental variables are not associated with any confounders of the exposure and outcome. Lastly, the effects of instrumental variables on the outcome are only through the exposure of interest (Burgess and Thompson, 2017).

### Statistical Analysis

In total, these 20 SNPs for telomere length were merged with the genetic associations (effect sizes and standard errors) of them from the AD GWAS^2^ following the guideline of performing MR analysis. The magnitudes (effect sizes) of the causal effects (odds ratio and 95% confidence interval) were estimated by applying various MR estimators. We used the inverse variance weighted method as our primary analysis. We treated the weighted median approach, MR-Egger regression, and weighed mode approaches as secondary and sensitivity analysis. This study only uses summary statistics, all data are publicly available, and no individual participant data were used, and an institutional ethical permit is therefore not necessary. These statistical analyses in this study were conducted from the *TwoSampleMR* package in R 4.0 (R Project for Statistical Computing). We also performed *E*-value to assess the minimum strength of association on the risk ratio scale that an unmeasured confounder would need to have with both the exposure and the outcome, conditional on the measured covariates, to fully explain away a specific exposure-outcome association. The input associations were obtained from a meta-analysis of case–control studies and two cohort studies, and analyses were performed using the online *E*-value calculator (Mathur et al., 2018).

## Results

**FIGURE 1:**
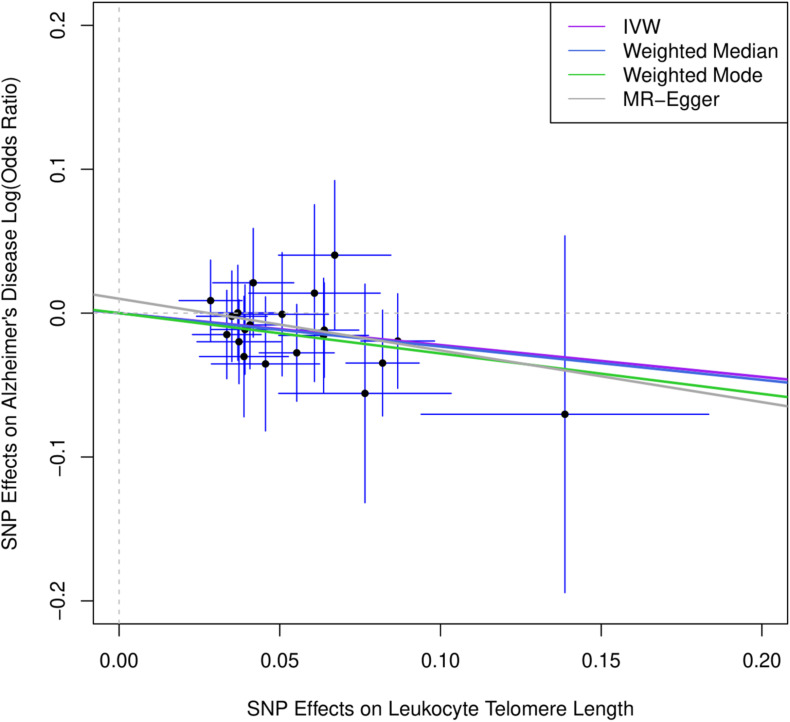
Scatter plot for genetic associations of SNPs on leukocyte telomere length and AD. IVW, inverse-variance weighted; MR, Mendelian randomization. Horizontal axis represents genetic associations of each genetic variant on leukocyte telomere length, and vertical axis denotes genetic associations of each genetic variant on AD.

**FIGURE 2:**
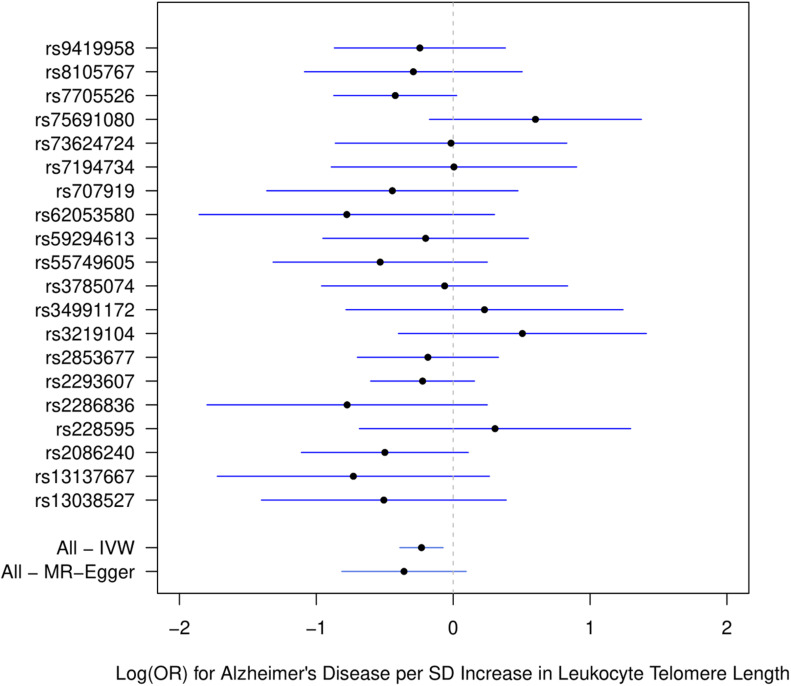
Single SNP plot for effects of SNPs on leukocyte telomere length and AD. IVW, inverse-variance weighted.

**FIGURE 3:**
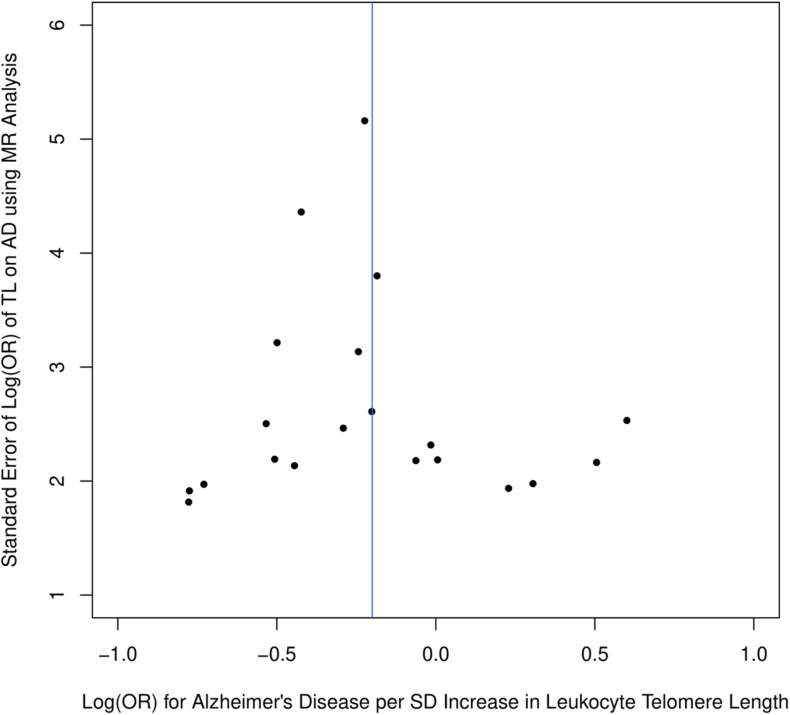
Funnel plot for selected SNPs as instrumental variables.

Figure 1 describes the genetic associations of the instrumental variables (genetic variants) on telomere length and AD. The causal effects of telomere length and AD were also presented. The results show that longer telomere length was associated with a lower risk of AD in almost all these different methods. The odds ratio was 0.79 (95% confidence interval: 0.67, 0.93, *P* = 0.004) for the inverse variance-weighted approach. The unit of this association was one standard deviation increase of telomere length. Likewise, the remaining MR methods, including the weighted median approach, MR-Egger regression, and weighted mode approaches, yielded very comparable point estimates. The minor differences were the confidence intervals, which are also comparable (Table 2). The intercept of the MR-Egger regression was not different from zero (β = −0.001, 95% CI: −0.03, 0.05, *P* = 0.62), which implies that a potential horizontal pleiotropy may not be a big concern. Further, no strong evidence was observed for the heterogeneity of these SNPs (*P* = 0.71). Similar results were obtained in the single SNP plot (Figure 2), which showed that no single SNP could affect the results noticeably. The funnel plot in Figure 3 implied that no single SNP might be an outlier or have a heterogeneous effect on the final MR estimations.

**TABLE 2:** Estimated causal effects of telomere length on Alzheimer’s disease using MR analysis.

Methods	OR	95% CI
Inverse variance weighted	0.79	0.68, 0.93
MR-Egger regression	0.70	0.44, 1.10
Weighted median	0.79	0.63, 0.99
Weighted mode	0.76	0.58, 0.99

*MR, Mendelian randomization.*

## Discussion

In the present study, we analyzed the GWAS summary statistics using the MR design and various estimation methods to study the relationship between leukocyte telomere length and AD, involving 35,274 individuals with AD and 59,163 controls. By taking advantage of 20 SNPs as instrumental variables, we found that longer telomere length could reduce the risk of AD. This study, updating previous studies (Zhan et al., 2015; Gao et al., 2019; Guo and Yu, 2019), represents the largest MR study to date on telomere length and clinically diagnosed AD, further contributing to the knowledge of AD etiology and suggesting that telomeres may be of paramount importance in AD pathogenesis.

Previously, a meta-analysis summarized the published studies on the topic of telomere length and AD. In this study, it showed that patients with AD might have shorter telomere length compared with controls (Forero et al., 2016). However, this study did not mention what confounders each of these studies had controlled for. Therefore, the residual confounding could explain the reported results. Our current study, on the other hand, can address the concern of unmeasured or uncontrolled confounding by re-analyzing GWAS summary statistics, which are of large sample size and well-conducted. However, our analysis is of limitations as well. MR analysis relies on three assumptions. In particular, the third assumption that there is no pleiotropic effect means that the genetic variants (SNPs and genes) selected as instrumental variables have effects on the outcome (e.g., AD) only through the exposure, which is the main interest (e.g., telomeres). This assumption, however, cannot be tested in practice. Most of the time, it relies on biological knowledge. We performed the MR-Egger regression test and found that the intercept of the MR-Egger regression is close to zero, implying that evidence of pleiotropy is not strong. The small discrepancy between MR-Egger estimates and other MR estimates may lie in the fact that the statistical power of MR-Egger is known to be lower than that of inverse-variance weighted. Therefore, the MR-Egger regression method is usually used to assess the pleiotropy assumptions by its intercept.

We additionally performed the calculation of *E*-value defined as the minimum strength of association on the risk ratio scale that an unmeasured confounder would need to have with both the exposure and the outcome, conditional on the measured covariates, to fully explain away a specific exposure-outcome association. The results implied that the unmeasured confounding must be strong enough to explain away the observed association in the epidemiological studies (Supplementary Table 2).

The exact biological mechanisms of the observed association between leukocyte telomere length and AD are to be explored. Several potential pathways could be proposed. Firstly, previous studies suggested that telomere maintained genomic stability and played a role in neuroplasticity to oxidative stress (Zhang et al., 2007; Spilsbury et al., 2015). In the telomerase RNA component (*TERC*) knockout mice model, neuronal loss in the frontal cortex was observed (Rolyan et al., 2011). Secondly, shortened telomeres were correlated with amyloid plaques in the transgenic mice (Baruch-Eliyahu et al., 2019). As oxidative stress and inflammation are more pronounced in the elderly, these mechanisms could be related to both telomeres’ shortening and aging manifestations such as cognitive disorders (Eitan et al., 2014). Thirdly, proinflammatory biomarkers, tumor necrosis factor-α, were reported to be associated with both telomeres and downstream senescence in microglial cells (Panossian et al., 2003). The activation of microglial cells could further lead to changes in the immunological microenvironment and AD progression (Hansen et al., 2018; Barak et al., 2020; Czako et al., 2020; Levit et al., 2020; Nguyen et al., 2020).

Although this is the largest MR analysis on telomere length and AD, the full summary statistics of the most recent GWAS of telomere length was unavailable. This limitation prevents us from performing further functional genomic analysis using GWAS summary statistics.

## Conclusion

Our present analyses applied an MR approach and found additional evidence for a causal relationship between telomere length and AD. Further studies that focus on the elucidation of this association could provide pivotal insights into the physiological roles of telomeres in AD pathogenesis.

## Data Availability Statement

The original contributions presented in the study are included in the article/Supplementary Material, further inquiries can be directed to the corresponding authors.

## Author Contributions

LL and ZM: conceptualization, writing – original draft preparation, and writing – review and editing. GY, LL, SW, and ZM: investigation. All authors have read and agreed to the published version of the article.

## Conflict of Interest

The authors declare that the research was conducted in the absence of any commercial or financial relationships that could be construed as a potential conflict of interest.

## Footnotes

1 https://downloads.lcbru.le.ac.uk/engage

2 https://www.niagads.org/datasets/ng00075
