# Supplementary material for: Genetically Predicted Telomere Length and Its Relationship With Alzheimer’s Disease
Source: Front Genet. 2021 Feb 19;12:595864. doi: 10.3389/fgene.2021.595864 (PMC7934420; doi:10.3389/fgene.2021.595864)
Supplement: Supplementary file 1 [file Data_Sheet_1.docx]

**STable 1.** Observational Studies on the Association between telomere length and Alzheimer’s disease

| **Author, Year** | **Country** | **Design** | **Sample Size**  **Cases/controls** | **% Men** | **Mean Ages** | **TL difference** | **OR/RR (95% CI)** |
| --- | --- | --- | --- | --- | --- | --- | --- |
| Fani, et al. 2020 | Netherlands | Cohort | 1961 | 43 | 71 |  | 1.59, 95% CI: 1.13, 2.23 |
| Tedone, et al. 2015 | Italy | Case-Contsrol | 31/20 | 41 | 80 | -0.42, 95%CI:-0.99, -0.14 |  |
| Kota, et al. 2015 | India | Case-Control | 57/55 | 48 | 65 | -0.67, 95%CI:-1.05,-0.29 |  |
| Mathur, et al. 2014 | Canada | Case-Control | 41/41 | 46 | 75 | -2.51, 95%CI:-3.09,-1.93 |  |
| Guan, et al. 2012 | Japan | Case-Control | 40/59 | 49 | 70 | -0.43, 95%CI:-0.84,-0.03 |  |
| Takata, et al. 2012 | Japan | Case-Control | 74/35 | 36 | 79 | -0.30, 95%CI:-0.70,-0.11 |  |
| Hochstrasser, et al. 2012 | Austria | Case-Control | 18/14 | 34 | 75 | -3.41, 95%CI:-4.51,-2.32 |  |
| Movérare-Skrtic, et al.2012 | Sweden | Case-Control | 32/20 | 48 | 75 | NA: only p-value was reported |  |
| Honig, et al.2012 | United States | Cohort | 1783 | 20 | 80 |  | 1.21, 95% CI: 1.00, 1.46 |
| Zekry, et al.2010 | Switzerland | Case-Control | 80/204 | NA | NA |  | 0.55, 95%CI: 0.30, 1.01 |
| Lukens, et al.2009 | United States | Case-Control | 29/22 | 35 | 80 | 0.23, 95%CI:-0.33,-0.79 |  |
| Thomas, et al.2008-1 | Australia | Case-Control | 54/26 | 36 | 75 | -1.05, 95%CI:-1.54,-0.56 |  |
| Thomas, et al. 2008-2 | Australia | Case-Control | 54/26 | 36 | 75 | -0.52, 95%CI:-0.99,-0.04 |  |
| Thomas, et al. 2008-3 | Australia | Case-Control | 13/9 | 48 | 76 | 1.20, 95%CI:0.28,2.12 |  |
| Franco, et al. 2006 | United States | Case-Control | 8/7 | 62 | 73 | -5.42, 95%CI:-7.63,-3.23 |  |
| Panossian, et al. 2003 | United States | Case-Control | 15/15 | 85 | 71 | -2.94, 95%CI:-3.97,-1.91 |  |

**STable 2.** E-value calculation

| **Study design** | **Reported association** | **E-value** |
| --- | --- | --- |
| Case-Control summary estimate (Diego A Forero, et al. 2016) | -0.984, 95% CI :−1.433, −0.535 | 4.33 |
| Cohort Estimate 1 (Fani, et al. 2020) | 1.59, 95% CI: 1.13, 2.23 | 2.56 |
| Cohort Estimate 2 (Fani, et al. 2020) | 1.47, 95% CI: 1.03, 2.10 | 2.30 |
| Cohort Estimate 3 (Honig, et al.2012) | 1.21, 95% CI: 1.00, 1.46 | 1.71 |

E-value: the minimum strength of association on the risk ratio scale that an unmeasured confounder would need to have with both the exposure and the outcome, conditional on the measured covariates, to fully explain away a specific exposure-outcome association.

**STable 3 Comparing with previous IVs**

| **Chr** | **Position (hg37)** | **SNP** | **Genes** | **SNPs selected as IV in Zhan, et al 2015** | **SNPs used in both studies** |
| --- | --- | --- | --- | --- | --- |
| 1 | 226562621 | rs3219104 | PARP1 |  |  |
|  |  |  |  | rs11125529 (chr2:54475866), *ACYP2* |  |
| 3 | 101232093 | rs55749605 | SENP7 |  |  |
| 3 | 169482335 | rs2293607 | TERC | rs10936599 (*r^2^*=1) | Yes |
| 4 | 71774347 | rs13137667 | MOB1B |  |  |
| 4 | 164098317 | rs2086240 | NAF1 | rs7675998(*r^2^*=1) | Yes |
| 5 | 1285974 | rs7705526 | TERT | rs2736100(*r^2^*=1) | Yes |
| 5 | 1287194 | rs2853677 | TERT |  |  |
| 6 | 25480328 | rs34991172 | CARMIL1 |  |  |
| 6 | 31641139 | rs707919 | PRRC2A |  |  |
| 7 | 124554267 | rs59294613 | POT1 |  |  |
| 10 | 105675946 | rs9419958 | OBFC1 | rs9420907(*r^2^*=1) | Yes |
| 11 | 108105593 | rs228595 | ATM |  |  |
| 14 | 73442192 | rs2286836 | DCAF4 |  |  |
| 16 | 82199980 | rs7194734 | MPHOSPH6 |  |  |
| 16 | 69406986 | rs3785074 | TERF2 |  |  |
| 16 | 74680074 | rs62053580 | RFWD3 |  |  |
| 19 | 22215441 | rs8105767 | ZNF208 | rs8105767(*r^2^*=1) | Yes |
| 20 | 62269750 | rs75691080 | RTEL1 | rs755017(*r^2^*=1) | Yesss |
| 20 | 62436398 | rs73624724 | RTEL1 |  |  |
| 20 | 62218340 | rs13038527 | RTEL1 |  |  |

**STable 4 Previous MR studies on this topic**

| **Studies** | **Number of SNPs** | **AD assessment** | **Sample size** |
| --- | --- | --- | --- |
| **Zhan, et al 2015** | **7** | **Clinically diagnosed** | **17008 AD +37054 controls** |
| **Guo, et al 2019** | **7** | **Proxy-AD** | **71880 AD + 383378 controls** |
| **Gao, et al 2019** | **16** | **Proxy-AD** | **71880 AD + 38337ss8 controls** |
| **Our study** | **20** | **Clinically diagnosed** | **35274 AD + 59163 controls** |

###################################

# main R codes for the MR analysis

library(data.table)

library(dplyr)

library(TwoSampleMR)

exposure_temp <- telomere20ad %>% transmute(

snp = snp, ea = ea, oa=oa, betax = beta, sex = se, px = pvalue, eaf = eaf, n=n_samples

)

telomere20_exp_dat <- format_data(exposure_temp, type = "exposure", snp_col = "snp",

beta_col = "betax", se_col = "sex", eaf_col = "eaf",

effect_allele_col = "ea", other_allele_col = "oa",

pval_col = "px"

)

outcome_temp <- telomere20ad %>% transmute(

snp = snp, ea = Effect_allele, oa = Non_Effect_allele, betay = Beta, sey = SE, py = Pvalue

)

telomere20_out_dat <- format_data(outcome_temp, type = "outcome", snp_col = "snp",

beta_col = "betay", se_col = "sey", eaf_col = "eaf",

effect_allele_col = "ea", other_allele_col = "oa",

pval_col = "py", ncase_col = "ncase", ncontrol_col = "ncontrol"

)

dat <- harmonise_data(

exposure_dat = telomere20_exp_dat,

outcome_dat = telomere20_out_dat, action = 2

)

head(dat)

res <- mr(dat)

res

cbind(

exp(res$b),

exp(res$b - 1.96 * res$se),

exp(res$b + 1.96 * res$se)

)

mr_heterogeneity(dat)

mr_pleiotropy_test(dat)

res <- mr(dat, method_list = c("mr_ivw", "mr_egger_regression", "mr_weighted_median", "mr_weighted_mode"))

p1 <- mr_scatter_plot(res, dat)

p1

ggplot2::ggsave(p1[[1]], file = "ScatterPlot.pdf", width = 7, height = 7)

res_single <- mr_singlesnp(dat)

p2 <- mr_forest_plot(res_single)

p2[[1]]

ggplot2::ggsave(p2[[1]], file = "SingleSNP_Plot.pdf", width = 7, height = 7)

res_loo <- mr_leaveoneout(dat)

res_loo

p3 <- mr_leaveoneout_plot(res_loo)

ggplot2::ggsave(p3[[1]], file = "LeaveOneOutPlot.pdf", width = 7, height = 7)

p4 <- mr_funnel_plot(res_single)

p4[[1]]

ggplot2::ggsave(p4[[1]], file = "FunnelPlot.pdf", width = 7, height = 7)

mr_report(dat)

#####################################
